# Design and cutting performance analysis of cylindrical gear skiving tool with uniform working rake angle

**DOI:** 10.1038/s41598-026-40178-2

**Published:** 2026-02-18

**Authors:** Jiaxue Ji, Peng Wang, Rui Xue, Tiegang Wang, Kan Xing, Jiawei Li

**Affiliations:** 1 Tianjin High-end Intelligent Machine Tool Engineering Research Center, Tianjin University of Technology and Education, Tianjin, 300222 China; 2 Postdoctoral Research Station in Mechanical Engineering, Tianjin University, Tianjin, 300182 China; 3 Genertec Tianjin No.1 Machine Tool Postdoctoral Research Center, Tianjin, 300385 China; 4 Tianjin Enterprise Key Laboratory of Automotive Synchronizer, Tianjin TANHAS Technology Co., Ltd., Tianjin, 301600 China; 5 Tianjin No. 1 Machine Tool Co., Ltd., Tianjin, 300385 China

**Keywords:** Gear skiving, Cylindrical skiving tool, Origin offset, Surface conjugation, Uniform working rake angle, Engineering, Mathematics and computing

## Abstract

Gear skiving is an efficient and precise method for gear manufacturing. Traditional conical skiving tools feature a structural relief angle on the flank and a plane rake face, which often leads to inconsistent accuracy after regrinding and an unreasonable working rake angle. This paper proposes a novel design method for cylindrical gear skiving tool with uniform working rake angle based on origin offset. A motion model for offset gear skiving is established, and the conjugate contact relationship between the tool and the workpiece is derived. The cutting tool is constructed by a free-form rake face and a helical flank face. The working rake angle is controlled to be uniform, and the flank face is confirmed to have no interference through sweeping trajectory calculation. A multi-physics coupling simulation model for cutting forces and temperature field is developed using finite element method. The results demonstrate that, compared to the conventional plane-rake-face tool, the proposed tool with a curved-rake-face significantly reduces cutting force fluctuations and peak temperature, leading to enhanced tool life, improved machining stability, and superior gear accuracy. A cutting experiment verified the correctness and effectiveness of the proposed tool design method.

## Introduction

Gear skiving demonstrates high machining precision and remarkable efficiency in processing complex gear structures in industrial products. In recent years, gear skiving has attracted significant attention from global research institutions and industrial enterprises. The structural design of skiving tool is a focal point in both theoretical and applied research as one of the critical factors influencing machining performance and tool longevity. However, conventional skiving tools, particularly those with conical geometry and a plane rake face, exhibit inherent limitations that hinder their performance and longevity. The existing skiving cutters often have non-uniform and even negative rake angles and unreasonable clearance angles resulting in rapid wear and low service life that cannot meet the engineering requirements. More specifically, the planar rake face, when combined with the offset machining conditions often employed to generate clearance angles, leads to significant variation in the local effective rake angle along the cutting edge. This inconsistency can result in poor chip formation, elevated and fluctuating cutting forces, and concentrated stress and thermal loads, which collectively accelerate tool wear and compromise machining stability and surface finish. Furthermore, the flank face of traditional tools often incorporates a structural relief angle, which is diminished upon regrinding, leading to a loss of machining accuracy after each resharpening cycle and increasing tooling costs over its lifecycle.

To overcome these drawbacks, this paper proposes a novel cylindrical gear skiving tool design based on the surface conjugation principle with an origin offset. The core innovation lies in the construction of a free-form curved rake face dynamically adjusted to maintain a uniform working rake angle along the entire cutting edge under offset machining conditions. This design fundamentally addresses the issue of inconsistent cutting geometry inherent in plane rake face tools. The primary advantages of the proposed design are multifaceted: (1) Enhanced Cutting Stability: The uniform rake angle promotes consistent chip formation and flow, leading to a significant reduction in cutting force fluctuations and cyclic impact loading, thereby improving process stability and protecting the tool from fatigue failure. (2) Improved Thermal Management: The optimized tool geometry facilitates better heat distribution, lowering the peak cutting temperature and creating a more gradual thermal gradient. This reduces the risk of thermal cracking and diffusion wear, contributing to extended tool life. (3) Maintained Accuracy After Regrinding: The flank face is designed as a cylindrical helical surface without a structural relief angle. This allows the tool to be reground solely on the rake face without altering the conjugate cutting edge geometry or the clearance provided by the offset machining kinematics, thereby preserving machining accuracy throughout the tool’s service life. (4) Interference-Free Operation: The helical flank face, combined with the origin offset method, is analytically verified through a sweeping trajectory model to ensure no interference occurs between the tool and the machined gear tooth, guaranteeing process reliability.

In order to enhance the wear resistance and service life of gear skiving tools, the cutting mechanism of gear skiving has been studied, and several innovative tool geometry design methods have been developed. Ichiro Moriwaki et al.^1^ systematically analyzed the effects of machining parameters, machine adjustments, and tool geometries on tooth surface characteristics, establishing guidelines for optimal tool angles. Bruno Vargas et al.^2,3^ highlighted the complex transient cutting conditions in skiving, particularly localized negative rake angles, significantly affect cutting forces. By integrating the Kienzle mechanical model, the calculation of instantaneous force achieved high accuracy to reflect the influence of rake angle. Pierce McCloskey et al.^4^ employed Delaunay triangulation to analyze chip cross-sections and compute cutting forces based on oblique cutting models. Hideaki Onozuka et al.^5^ developed a micro-element-based force model incorporating cutting velocity, chip thickness, and effective rake angles, reducing prediction percentage error to below 15%. Ren Zongwei et al.^6^ numerically predicted crater wear on rake faces by analyzing chip morphology, tool angles, and cutting depth. These studies indicate the necessity of innovative design for the structure of skiving tools. Li Jia et al.^7,8^ proposed a free-form cutting edge and surfaces configuration method according to the surface conjugate principle under the design philosophy of uniform working rake angle and consistent regrinding accuracy. Jia Kang^9^ established a precise model to analyse the complex skiving interactions integrating contact point determination and spatial skiving motion simulation. Chung-Yu Tsai et al.^10,11^ proposed a cylindrical skiving tool design method by introducing an inclination angle to avoid tool interference in skiving processing. Yi-Pei Shih et al. ^12^ construct the cylindrical skiving tool by offsetting the rake face by an appropriate distance to generate clearance angle. Guo Erkuo et al.^13,14^ proposed an optimization method for cylindrical tool to eliminate interference and balance the side clearance angle by adjusting the axial offset and helix angle, and calculated the cutting force based on undeformed chips. Zhao Shuaijie et al.^15^ provided a comprehensive review of gear skiving technology, critically analyzing its principles, challenges, and future directions to enhance machining efficiency and precision.

Although significant advancements have been made in the design of skiving tools, the existing technologies still face bottlenecks such as regrinding-induced accuracy loss and uneven wear. The common gear skiving tools predominantly adopt a planar-involute retracting surface, which results in poor cutting performance. To address these issues, this paper proposes a novel design method for cylindrical gear skiving tools based on the surface conjugation principle. The cutting edge is constructed as the conjugate line of the workpiece profile under the processing motion with origin offset. And then, a dynamically adjusted free-form rake face is estabished to enable uniform working rake angles. The flank face is generated by the cutting edge spiraling along the tool axis, and the helix angle is set based on the purpose of avoiding interference.

It is acknowledged that the high-precision manufacturing of the proposed free-form curved rake face presents certain challenges. The primary difficulties lie in accurately grinding the complex three-dimensional surface contour and ensuring the required surface finish. However, these challenges can be effectively addressed with modern advanced manufacturing techniques. Suitable machining processes include multi-axis computer numerical control grinding using a forming grinding wheel, whose profile is precisely dressed to match the desired rake face geometry. Alternatively, slow-tool-servo or fast-tool-servo turning on a precision lathe could be employed for generating such free-form surfaces on indexable inserts. The geometric data of the designed rake face can be directly used to program these advanced machines, ensuring manufacturing accuracy. Subsequent precision metrology, such as coordinate measuring machines or optical profilometers, can be used for verification.

A multi-physics coupling simulation of cutting force and temperature field is conducted by finite element method to elucidate the cutting mechanism and machining performance of the designed skiving tool. The cutting performance of the designed skiving tool with curved rake face was compared with that of the cutting tool with plane rake face. Finally, a cutting experiment was carried out to validate the engineering applicability of the proposed tool design method. This work could provide theoretical and technical support for high-precision and efficient gear machining.

## Skiving tool design

### Offset skiving motions

**Fig. 1:**
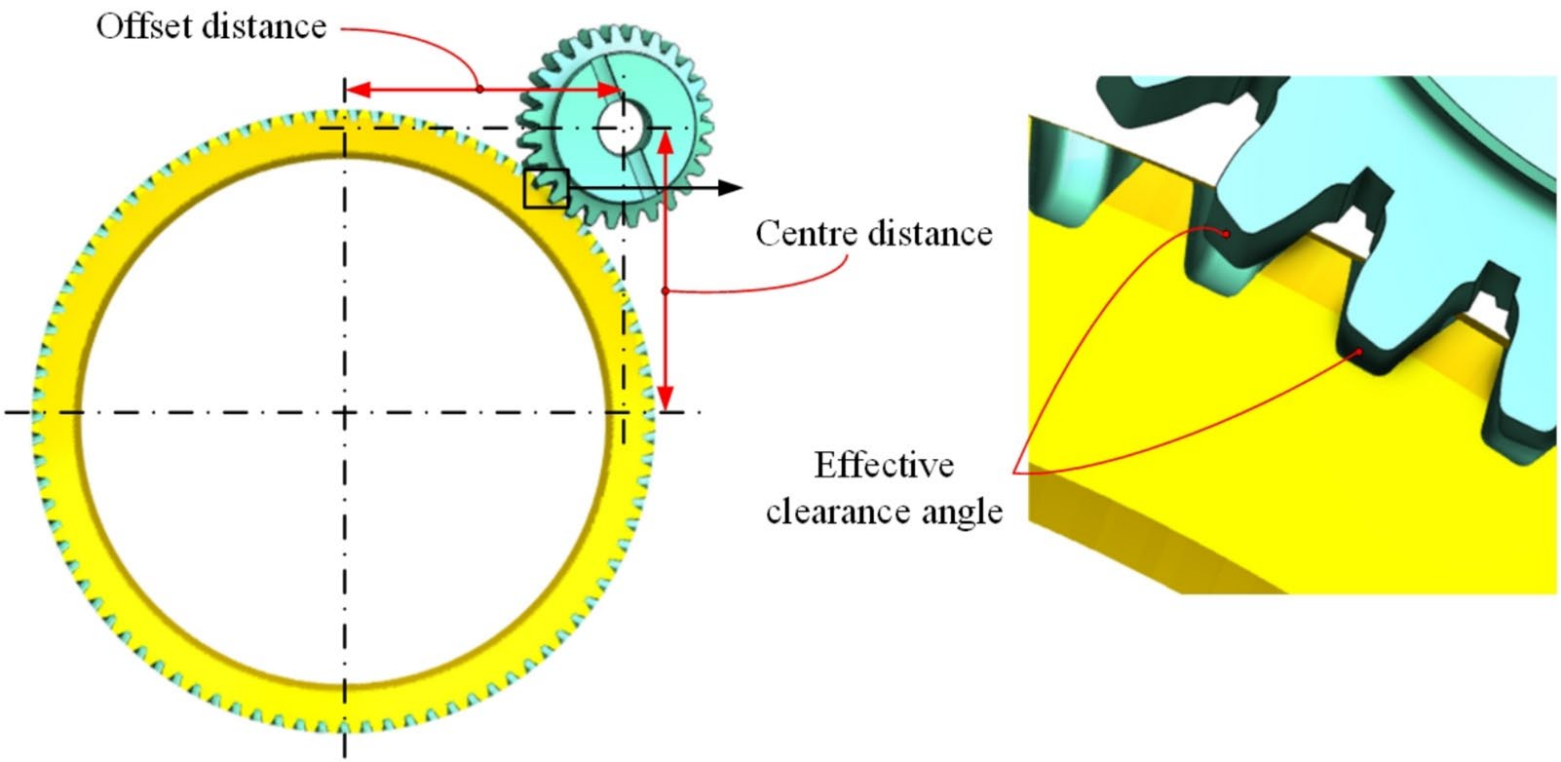
Offset gear skiving machining principle diagram.

To reduce the design and manufacturing difficulty of the skiving tool’s flank face and to form an effective working clearance angle, this study proposes a linear origin-offset-based skiving method, as shown in Fig. 1, on conventional machine tools. By linear offset perpendicular to the common center line of the tool and the workpiece and the center distance adjusting, a functional clearance angle can be generated during skiving motions.

**Fig. 2:**
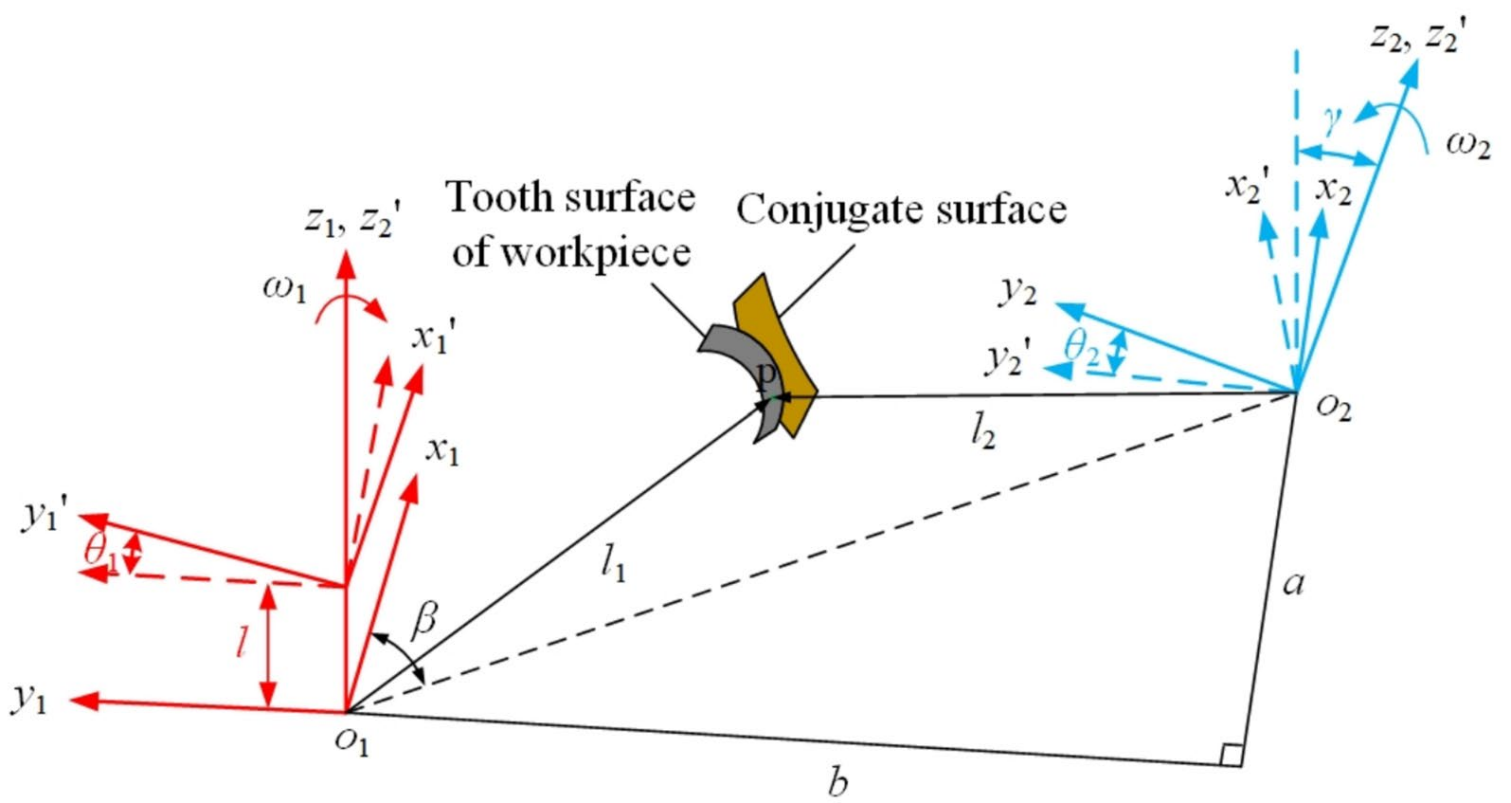
Four coordinate systems for the skiving motion model.

In order to explain the process of offset gear skiving, a coordinate system is established as shown in Fig. 2. At the initial moment, the *x*_1_-axis and *x*_2_-axis are parallel and aligned in the same direction. The coordinate system S_1_(*o*_1_, *x*_1_, *y*_1_, *z*_1_) is defined as the workpiece coordinate system, used to describe the tooth surface of the workpiece. This system is fixed to the workpiece, with the *z*_1_-axis oriented along the workpiece axis, and it moves according to the rotation and axial feed of the workpiece. The coordinate system S_2_(*o*_2_, *x*_2_, *y*_2_, *z*_2_) serves as the tool coordinate system, established for modeling the tool. The *z*_2_-axis is set as the tool axis, such that the angle between the *z*_2_-axis and the *z*_1_-axis equals the shaft intersection angle *γ*. Meanwhile, this coordinate system rotates about the *z*_2_-axis at the tool’s rotational speed. The coordinate systems S_1_' (*o*_1_', *x*_1_', *y*_1_', *z*_1_') and S_2_' (*o*_2_', *x*_2_', *y*_2_', *z*_2_') are used to describe the positions of the workpiece and the skiving tool after origin offset. The system S_1_' is fixed to the workpiece and moves with it, while S_2_' is fixed to the tool and rotates with it.

As shown in Fig. 2, a positive angular velocity is assigned to counterclockwise rotation and a negative value to clockwise rotation for both the skiving tool and the workpiece. In the depicted configuration, both the tool and the workpiece rotate in a left-handed direction about their respective *z*-axes. The relationship between their angular velocities is governed by the shaft intersection angle *γ* and the gear ratio, which is determined by the number of teeth on the workpiece (*N*_*w*_) and the tool (*N*_*t*_). For continuous generating motion, the ratio must satisfy:

1 $$\frac{{\omega_{2} }}{{\omega_{1} }} = \frac{{N_{w} }}{{N_{t} }}$$

Where $\omega_{1}$: Angular velocity of the workpiece about its axis (*z*_1_).$\omega_{2}$: Angular velocity of the tool about its axis (*z*_2_).

In the coordinate systems defined, with the given rotation directions and the shaft intersection angle *γ* (where *γ* < 0 in this setup), the corresponding angular velocity vectors are:

2 $$\left\{ \begin{gathered} {\boldsymbol{\omega}}_{1} = \omega_{1} {\boldsymbol{k}}_{1} \hfill \\ {\boldsymbol{\omega}}_{2} = \omega_{2} {\boldsymbol{k}}_{2} \hfill \\ \end{gathered} \right.$$

where ${\boldsymbol{k}}_{1}$ and ${\boldsymbol{k}}_{2}$ are the unit vectors along the *z*_1_-axis and *z*_2_-axis, respectively.

The transformation between coordinate systems follows the standard rule of rigid body kinematics: a point expressed in one coordinate system can be represented in another through a rotation followed by a translation. This is efficiently expressed using homogeneous transformation matrices.

At a certain moment, the workpiece rotates angle *θ*_1_ relative to the initial position, and feeds along its axis for distance *l* with coordinate system S_1_. the tool rotates angle *θ*_2_ relative to the initial position with coordinate system S_2_. The interaxial relationship between the two systems show as the included angle *γ* between axis *z*_1_ and axis *z*_2_, the center distance *a*, and the offset distance *b*. According to Fig. 2, the transformation matrix between the coordinate systems can be obtained, as shown in Eq. (3).

3 $$\begin{gathered} M_{22^{\prime}} = \left[ \begin{gathered} \cos \theta_{2} { - }\sin \theta_{{2}} \, 0 0 \, \hfill \\ \sin \theta_{2} \, \cos \theta_{{2}} \, 0 0 \hfill \\ \, 0 \, 0 1 \, 0 \hfill \\ \, 0 \, 0 0 \, 1 \hfill \\ \end{gathered} \right] \, M_{2^{\prime}1^{\prime}} = \left[ \begin{gathered} 1 \, 0 0 a \hfill \\ 0 \, \cos \gamma { - }\sin \gamma { - }b \hfill \\ 0 \, \sin \gamma \, \cos \gamma \, 0 \hfill \\ 0 \, 0 \, 0 \, 1 \hfill \\ \end{gathered} \right] \hfill \\ M_{1^{\prime}1} = \left[ \begin{gathered} \cos \theta_{1} \, \sin \theta_{{1}} \, 0 0 \, \hfill \\ { - }\sin \theta_{1} \, \cos \theta_{{1}} \, 0 0 \hfill \\ \, 0 \, 0 1 \, l \, \hfill \\ \, 0 \, 0 0 1 \hfill \\ \end{gathered} \right] \hfill \\ \end{gathered}$$

### Derivation of conjugate surface

In order to ensure the machining accuracy of the tooth profile, it is required that the cutting edge could conjugate with the tooth surface of the workpiece during the machining process. As shown in Fig. 2, the equation of the tooth surface is expressed in coordinate system S_1_, as Eq. (4).

4 $${\mathbf{L}}_{1} = {\mathbf{L}}_{1} (\theta ,\mu ) = x_{{1}} (\theta ,\mu ){\boldsymbol{i}}_{{1}} + y_{{1}} (\theta ,\mu ){\boldsymbol{j}}_{{1}} + z_{{1}} (\theta ,\mu ){\boldsymbol{k}}_{{1}}$$

Where ***i***_1_, ***j***_1_, ***k***_1_ represent the unit vectors of the coordinate axes *x*_1_, *y*_1_, *z*_1_ respectively, and *θ*, *μ* are the two parameters of the tooth surface.

Equation of the conjugate surface should be expressed in the coordinate system S_2_, as Eq. (5).

5 $${\mathbf{L}}_{2} = x_{2} {\boldsymbol{i}}_{2} + y_{2} {\boldsymbol{j}}_{2} + z_{2} {\boldsymbol{k}}_{2}$$

Where ***i***_2_, ***j***_2_, ***k***_2_ represent the unit vectors of the coordinate axes *x*_2_, *y*_2_, *z*_2_ respectively.

According to the fact that the workpiece is in contact with the conjugate surface at a certain point, the conjugate surface can be further transformed from S_1_ to S_2_ by the equation of the tooth surface. The transformation relationship is shown in Eq. (6).

6 $$\left[ \begin{gathered} x_{2} \\ y_{2} \\ z_{2} \\ 1 \\ \end{gathered} \right] = M_{21} \left[ \begin{gathered} x_{1} \\ y_{1} \\ z_{1} \\ 1 \\ \end{gathered} \right] \,$$

7 $$M_{21} = M_{22^{\prime}} M_{2^{\prime}1^{\prime}} M_{1^{\prime}1}$$

Where, *M*_21_ is the coordinate transformation matrix, which can be obtained by Eq. (7). Then, the conjugate surface is derived as Eq. (8).

8 $$\left\{ \begin{gathered} x_{2} = x_{1} (\theta ,\mu )(\cos \theta_{1} \cos \theta_{2} + \sin \theta_{1} \sin \theta_{2} \cos \gamma ) + y_{1} (\theta ,\mu )( - \sin \theta_{1} \cos \theta_{2} + \cos \theta_{1} \hfill \\ \, \sin \theta_{2} \cos \gamma ) - z_{1} (\theta ,\mu )\sin \theta_{2} \sin \gamma + l\sin \theta_{2} \sin \gamma + a\cos \theta_{2} + b\sin \theta_{2} \cos \gamma \hfill \\ y_{2} = x_{1} (\theta ,\mu )( - \cos \theta_{1} \sin \theta_{2} + \sin \theta_{1} \cos \theta_{2} \cos \gamma ) + y_{1} (\theta ,\mu )(\sin \theta_{1} \sin \theta_{2} + \cos \theta_{1} \hfill \\ \, c{\mathrm{os}}\theta_{2} \cos \gamma ) - z_{1} (\theta ,\mu )\cos \theta_{2} \sin \gamma + l\cos \theta_{2} \sin \gamma - a\sin \theta_{2} + b\cos \theta_{2} \cos \gamma \hfill \\ z_{2} = x_{1} (\theta ,\mu )(\sin \theta_{1} \sin \gamma ) + y_{1} (\theta ,\mu )(\cos \theta_{1} \sin \gamma ) + z_{1} (\theta ,\mu )\cos \gamma - l\cos \gamma \hfill \\ \end{gathered} \right.$$

Where *θ*_1_, *θ*_2_ and *l* are the instantaneous motion variables of the workpiece rotation angle, tool rotation angle, and the axial displacement of the workpiece, respectively. These variables are linked through the machining kinematics. The parameter *f* is defined as the axial feed rate per revolution of the workpiece, with a unit of mm/r. It represents the axial distance the workpiece advances along its own axis (*z*_1_) for each complete revolution. During the continuous skiving process, the workpiece rotates with a constant angular velocity $\omega_{1}$ (rad/s). Therefore, the relationship between the axial displacement *l* and the workpiece rotation angle *θ*_1_ is linear and can be expressed by the following equation, which is crucial for linking rotational motion to linear feed:

9 $$l = \left( {\frac{f}{2\Pi }} \right) \cdot \theta_{1} = f \cdot \frac{{\theta_{1} }}{{\omega_{1} }} \cdot \frac{{\omega_{1} }}{2\Pi }$$

Given that $\omega_{1}$ is constant, this simplifies to the more direct form used in the kinematic derivation:

10 $$l = f\frac{{\theta_{1} }}{{\omega_{1} }}$$

This equation governs the synchronous motion between rotation and feed. The term (*θ*_1_/$\omega_{1}$) represents the elapsed machining time. Multiplying this time by the axial feed rate *f* yields the total axial feed displacement *l*. This coupling is essential for ensuring that the tool follows the prescribed helical path relative to the workpiece, generating the desired gear tooth geometry.

The kinematic conjugation principle dictates that the common normal vector ***n*** maintains orthogonality with the relative motion velocity ***v***_21_, as shown in Eq. (11).

11 $${\boldsymbol{n}} \cdot {\boldsymbol{v}}_{21} = 0$$

The expression of vector ***n***, in the coordinate system S_1_, can be obtained:

12 $$\begin{gathered} {\boldsymbol{n}} = \frac{{\partial {\boldsymbol{l}}_{1} }}{\partial \theta } \times \frac{{\partial {\boldsymbol{l}}_{1} }}{\partial \mu } = \left| \begin{gathered} {\boldsymbol{i}}_{1} \, {\boldsymbol{j}}_{{1}} \, {\boldsymbol{k}}_{{1}} \, \hfill \\ \frac{{\partial x_{1} }}{\partial \theta } \, \frac{{\partial y_{1} }}{\partial \theta } \, \frac{{\partial z_{1} }}{\partial \theta } \hfill \\ \frac{{\partial x_{1} }}{\partial \mu } \, \frac{{\partial y_{1} }}{\partial \mu } \, \frac{{\partial z_{1} }}{\partial \mu } \hfill \\ \end{gathered} \right| \hfill \\ \, = n_{x} {\boldsymbol{i}}_{1} + n_{y} {\boldsymbol{j}}_{1} + n_{z} {\boldsymbol{k}}_{1} \hfill \\ \end{gathered}$$

Where *n*_x_, *n*_y_, *n*_z_ are the component of ***n*** along coordinate axes *x*_1_, *y*_1_, *z*_1_.

As shown in Fig. 2, based on the position and motion relationships between the cutting tool and the workpiece, the speed of the tool relative to the workpiece at the conjugate point P can be obtained by Eq. (13).

13 $${\boldsymbol{v}}_{21} = {\boldsymbol{\omega}}_{2} \times {\boldsymbol{l}}_{2} - {\boldsymbol{\omega}}_{1} \times {\boldsymbol{l}}_{1} - {\boldsymbol{f}}$$

Where,

14 $$\left\{ \begin{gathered} {\boldsymbol{\omega}}_{{1}} = \omega_{{1}} {\boldsymbol{k}}_{1} \hfill \\ {\boldsymbol{\omega}}_{2} = \omega_{2} {\boldsymbol{k}}_{2} \hfill \\ {\boldsymbol{v}}_{f} = f{\boldsymbol{k}}_{1} \hfill \\ {\boldsymbol{l}}_{1} = x_{1} {\boldsymbol{i}}_{1} + y_{1} {\boldsymbol{j}}_{1} + z_{1} {\boldsymbol{k}}_{1} \hfill \\ {\boldsymbol{l}}_{2} = \overrightarrow {{{\rm O}_{2} ^{\prime}{\rm O}_{1} ^{\prime}}} + {\boldsymbol{l}}_{1} = ( - \sqrt {a^{2} + b^{2} } {)}\user2{ i}_{1} ^{\prime} + x_{1} {\boldsymbol{i}}_{1} + y_{1} {\boldsymbol{j}}_{1} + z_{1} {\boldsymbol{k}}_{1} \hfill \\ \end{gathered} \right.$$

According to the vector positional relationship shown in Fig. 2, the following equations can be obtained:

15 $$\left\{ \begin{gathered} {\boldsymbol{k}}_{2} = {\boldsymbol{i}}_{1} \sin \theta_{1} \sin \gamma + {\boldsymbol{j}}_{1} \cos \theta_{1} \sin \gamma + {\boldsymbol{k}}_{1} \cos \gamma \hfill \\ {\boldsymbol{i}}_{1} ^{\prime} = {\boldsymbol{i}}_{1} \cos \theta_{1} - {\boldsymbol{j}}_{1} \sin \theta_{1} \hfill \\ \end{gathered} \right.$$

By substituting Eq. (14) and Eq. (15) into Eq. (13), the expression of the relative motion velocity ***v***_21_ is derived as presented in Eq. (16).

16 $$\begin{gathered} \user2{ v}_{21} = v_{x} {\boldsymbol{i}}_{1} + v_{y} {\boldsymbol{j}}_{1} + v_{z} {\boldsymbol{k}}_{1} \hfill \\ \left\{ \begin{gathered} v_{x} = z_{1} \omega_{2} \cos \theta_{1} \sin \gamma - \omega_{2} (y_{1} + \sqrt {a^{2} + b^{2} } \sin \theta_{1} )\cos \gamma + y_{1} \omega_{1} \hfill \\ v_{y} = (x_{1} - \sqrt {a^{2} + b^{2} } \cos \theta_{1} )\omega_{2} \cos \gamma - z_{1} \omega_{2} \sin \theta_{1} \sin \gamma - x_{1} \omega_{1} \hfill \\ v_{z} = y_{1} \omega_{2} \sin \theta_{1} \sin \gamma - x_{1} \omega_{2} \cos \theta_{1} \sin \gamma - f \hfill \\ \end{gathered} \right. \hfill \\ \end{gathered}$$

By substituting Eq. (12) and Eq. (16) into Eq. (11), the governing relationship is derived as follows:

17 $$\begin{gathered} \, U\sin \theta_{1} - V\cos \theta_{1} = W \hfill \\ \left\{ \begin{gathered} U = - n_{x} \sqrt {a^{2} + b^{2} } \omega_{2} \cos \gamma - n_{y} z_{1} \omega_{2} \sin \gamma + n_{z} y_{1} \omega_{2} \sin \gamma \hfill \\ V = - n_{x} z_{1} \omega_{2} \sin \gamma + n_{y} \sqrt {a^{2} + b^{2} } \omega_{2} \cos \gamma + n_{z} x_{1} \omega_{2} \sin \gamma \hfill \\ W = (n_{x} y_{1} - n_{y} x_{1} )(\omega_{2} \cos \gamma - \omega_{1} ) + n_{z} f \hfill \\ \end{gathered} \right. \hfill \\ \end{gathered}$$

Solving Eq. (17) yields:

18 $$\theta_{1} = \arcsin (\frac{{UW \pm V\sqrt {U^{2} + V^{2} - W^{2} } }}{{U^{2} + V^{2} }})$$

Substituting Eqs. (10) and (18) into Eq. (8) yields the conjugate surface expression, simplified as:

19 $${\boldsymbol{L}}_{2} = {\boldsymbol{L}}_{2} (\theta ,\mu ) = x_{{2}} (\theta ,\mu ){\boldsymbol{i}}_{{2}} + y_{2} (\theta ,\mu ){\boldsymbol{j}}_{2} + z_{2} (\theta ,\mu ){\boldsymbol{k}}_{2}$$

## Cylindrical skiving tool design

### Curved rake face

**Fig. 3:**
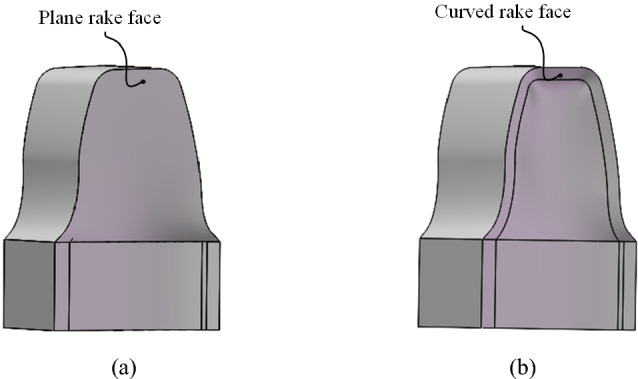
Rake face geometry variations.

The rake face of common skiving tool is usually a plane surface as shown in Fig. 3(a). Under the condition of offset machining, this type of cutting face has inconsistent and even negative rake angles, which affects the cutting performance of the tool. Therefore, this article proposes a design method for rake face with equal working angles as shown in Fig. 3(b). The rake face is designed as a free curved surface extending from the cutting edge which should be the intersection curve between a plane and the conjugate surface. In coordinate system S_2_, the aforementioned plane can be calculated in the manner shown in reference^16^, it is simply expressed as:

20 $${\boldsymbol{n}}(\gamma_{\varepsilon } ) \, \cdot \, ({\boldsymbol{r}} - {\boldsymbol{r}}_{0} ) = 0$$

Where, *γ*_*ε*_ represents the inclination angle of the plane, ***n***(*γ*_*ε*_) represents the normal vector of the plane and ***r***_0_ represents the intersection point of the tooth top line of the conjugate surface and the coordinate plane *x*_2_o_2_*y*_2_.

The cutting edge can be obtained by intersection of Eq. (8) and Eq. (20), expressed as:

21 $${\boldsymbol{r}} = x_{{\mathrm{r}}} {\boldsymbol{i}}_{{2}} + y_{{\mathrm{r}}} {\boldsymbol{j}}_{{2}} + z_{{\mathrm{r}}} {\boldsymbol{k}}_{{2}}$$

(a) Plane rake face (b) Curved rake face

**Fig. 4:**
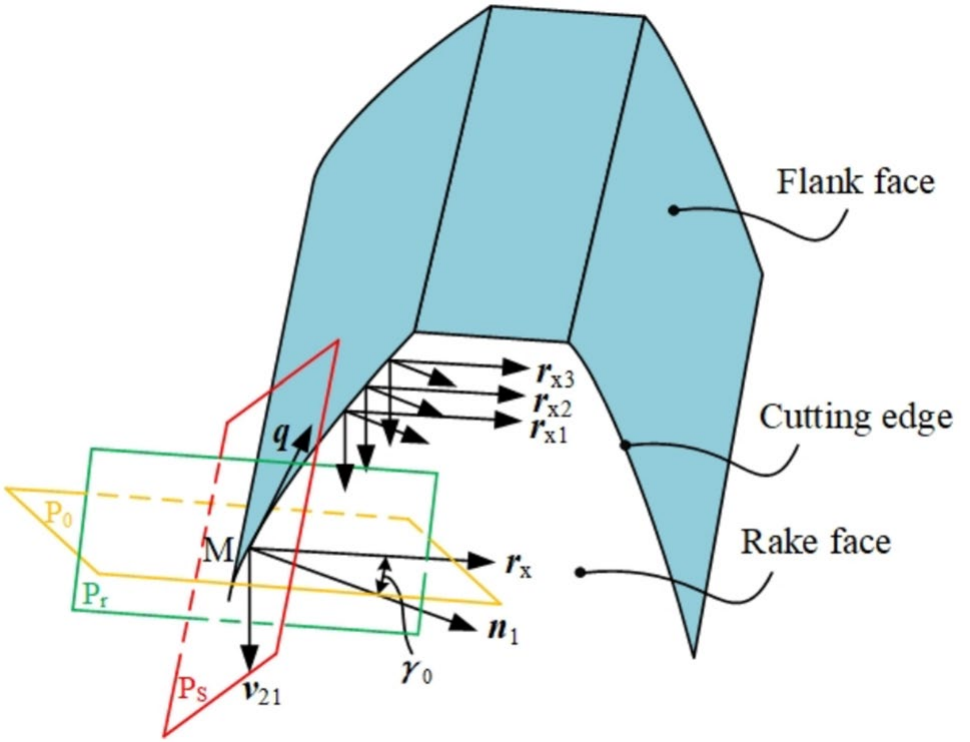
Cutting angle reference system.

As shown in Fig. 4, the curved rake face is formed by adjustment of working rake angle guided by cutting velocity and reference frames (base plane P_r_, cutting plane P_s_, and orthogonal plane P_o_).

As shown in Fig. 4, an arbitrary point M on the cutting edge is analyzed. The cutting velocity ***v***_21_(*v*_x_, *v*_y_, *v*_z_) is derived from Eq. (16), while the tangent vector ***q***(*q*_x_, *q*_y_, *q*_z_) is obtained via first-order differentiation of the cutting edge equation. The normal vector ***n***_1_ to the cutting plane P_s_ at point M is determined by the cross product of ***q*** and ***v***_21_, expressed as:

22 $${\boldsymbol{n}}_{1} = {\boldsymbol{q}} \times {\boldsymbol{v}}_{21} = \left| \begin{gathered} {\boldsymbol{i}}{\text{ j k}} \hfill \\ q_{x} \, q_{y} \, q_{z} \hfill \\ v_{x} \, v_{y} \, v_{z} \hfill \\ \end{gathered} \right|$$

According to the principle of metal cutting, the working rake angle is defined as the intersection angle between vector ***n***_1_ and vector ***r***_x_ which represents the direction of the intersection line of orthogonal plane P_o_ and rake face. Here, working rake angle *γ*_o_ is specified for every discrete point on the cutting edge. On orthogonal plane P_o_, a straight line is constructed over point M along vector ***r***_x_, and it satisfies Eq. (23):

23 $${\boldsymbol{n}}_{1} - \left| {{\boldsymbol{n}}_{1} } \right|\tan \gamma_{{0}} \frac{{{\boldsymbol{v}}_{{{21}}} }}{{\left| {{\boldsymbol{v}}_{{{21}}} } \right|}} = \left| {{\boldsymbol{n}}_{{1}} } \right|\sec \gamma_{{0}} {\boldsymbol{r}}_{{\mathrm{x}}}$$

The rake face with uniform working angles is synthesized by constructing a family of straight lines over several discrete points of the cutting edge, along the directions of vectors ***r***_x1_, ***r***_x2_, ***r***_x3_ and so on, who are specified the same value of *γ*_o_.

### Flank face

**Fig. 5:**
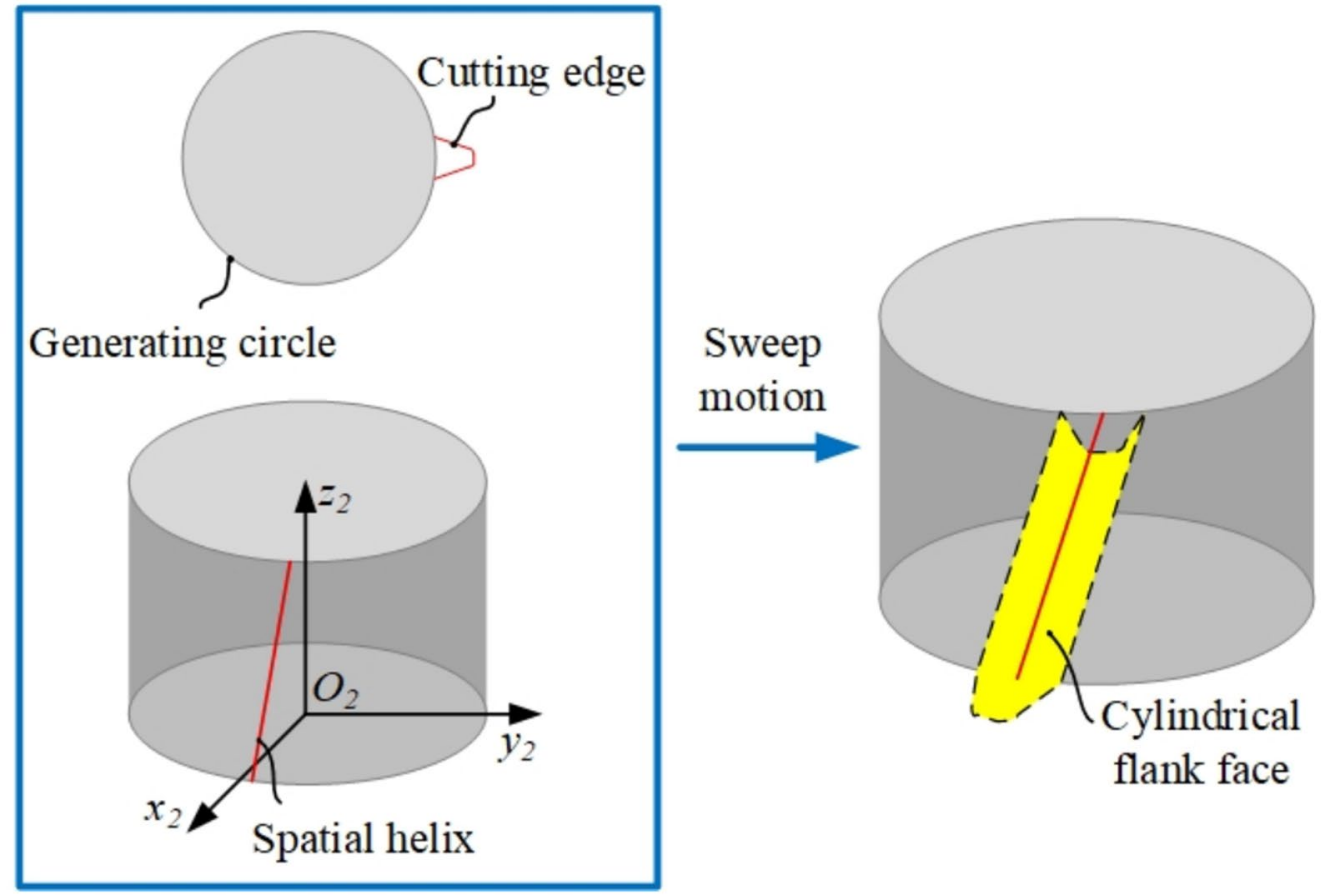
Schematic of cylindrical flank face.

The design of the flank face needs to take into account the convenience of tool manufacturing and regrinding, as well as the maintainability of processing accuracy, while avoiding any interference. For skiving tool with non-structured clearance angles, the flank face is constructed as a cylindrical helical surface on the basis of the cutting edge, as shown in Fig. 5.

The cylindrical flank face can be expressed as:

24 $$\left\{ {\begin{array}{*{20}c} {x_{R} = x_{r} \cos \theta - y_{r} \sin \theta } \\ {y_{R} = x_{r} \sin \theta + y_{r} \cos \theta } \\ {z_{R} = z_{r} + \frac{h}{2\pi }\theta } \\ \end{array} } \right.$$

Where, *θ* represents the helical surface parameter and *h* represents the helical pitch.

As shown in Fig. 5, the flank face is generated by spirally extending the cutting edge along the tool axis with a constant helical pitch. This cylindrical helical surface design is crucial as it ensures a constant clearance angle along the entire cutting edge, which is fundamental for simplifying the tool grinding process and maintaining consistent cutting performance after repeated regrinding.

The design of flank face centers on interference analysis between it and the machined surface. During the skiving process, the cutting edge initially approaches the workpiece, while the other parts of the flank face gradually approach the workpiece. The sweeping of the cutting edge cuts out a groove and forms the theoretical tooth surface. Therefore, when the sweeping paths of other parts of the flank face are included within the groove formed by the sweeping of the cutting edge, no interference will occur. This can serve as a criterion for determining whether there is interference or not. The model of sweeping trajectory is firstly established in the workpiece coordinate system S_1_. Let the processing time be *t*, and the trajectory surface is expressed as:

25 $${\boldsymbol{L}}_{{\mathrm{s}}} = {\boldsymbol{L}}_{{\mathrm{s}}} (\theta ,t) = x_{s} (\theta ,t){\boldsymbol{i}}_{{1}} + y_{s} (\theta ,t){\boldsymbol{j}}_{{1}} + z_{s} (\theta ,t){\boldsymbol{k}}_{{1}}$$

where, *x*_s_, *y*_s_, and *z*_s_ represent the coordinate components of one point on sweeping trajectory surface along axes *x*_1_, *y*_1_, and *z*_1_ respectively, and they are obtained by the following equation:

26 $$\left[ \begin{gathered} x_{s} \hfill \\ y_{s} \hfill \\ z_{s} \hfill \\ \, 1 \hfill \\ \end{gathered} \right] = M_{12}^{s} \left[ \begin{gathered} x_{r} \hfill \\ y_{r} \hfill \\ z_{r} \hfill \\ \, 1 \hfill \\ \end{gathered} \right]$$

where, *x*_r_, *y*_r_, and *z*_r_ represent the coordinate components of one point on the flank face along axes *x*_2_, *y*_2_, and *z*_2_ respectively, and $M_{12}^{s}$ is the coordinate transformation matrix corresponding to time *t*. With the aid of auxiliary coordinate systems S_1_' and S_2_', it can be obtained as:

27 $$M_{12}^{s} = M_{11^{\prime}}^{s} M_{1^{\prime}2^{\prime}}^{s} M_{2^{\prime}2}^{s}$$

In the equation,

28 $$\begin{gathered} M_{11^{\prime}}^{s} = \left[ \begin{gathered} \cos (\omega_{1} t){ - }\sin (\omega_{1} t) \, 0 0 \, \hfill \\ \sin (\omega_{1} t) \, \cos (\omega_{1} t) \, 0 0 \hfill \\ \, 0 \, 0 \, 1 { - }vt \hfill \\ \, 0 \, 0 \, 0 \, 1 \hfill \\ \end{gathered} \right] \, M_{1^{\prime}2^{\prime}}^{s} = \left[ \begin{gathered} 1 \, 0 0 { - }a \hfill \\ 0 \, \cos \gamma \, \sin \gamma \, b \hfill \\ 0{ - }\sin \gamma \, \cos \gamma \, 0 \hfill \\ 0 \, 0 \, 0 \, 1 \hfill \\ \end{gathered} \right] \hfill \\ M_{2^{\prime}2}^{s} = \left[ \begin{gathered} \cos (\omega_{2} t) \, \sin (\omega_{2} t) \, 0 0 \, \hfill \\ { - }\sin (\omega_{2} t) \, \cos (\omega_{2} t) \, 0 0 \hfill \\ \, 0 \, \, 0 \, 1 \, 0 \, \hfill \\ \, 0 \, \, 0 \, 0 1 \hfill \\ \end{gathered} \right] \hfill \\ \end{gathered}$$

From Eqs. (25)-(28), the parametric expression of sweeping trajectory surface is obtained as:

29 $$\left\{ \begin{gathered} x_{s} = x_{r} (\theta )[\cos (\omega {}_{1}t)\cos (\omega_{2} t) + \sin (\omega_{1} t)\sin (\omega_{2} t)\cos \gamma ] + y_{r} (\theta )[ - \cos (\omega_{1} t)\sin (\omega_{2} t) + \sin (\omega_{1} t) \hfill \\ \, \cos (\omega_{2} t)\cos \gamma ] + z_{r} (\theta )\sin (\omega_{1} t)\sin \gamma - a\cos (\omega_{1} t) - b\sin (\omega_{1} t)\cos \gamma \hfill \\ y_{s} = x_{r} (\theta )[ - \sin (\omega_{1} t)\cos (\omega_{2} t) + \cos (\omega_{1} t)\sin (\omega_{2} t)\cos \gamma ] + y_{r} (\theta )[\sin (\omega_{1} t)\sin (\omega_{2} t) + \cos (\omega_{1} t) \hfill \\ \, \cos (\omega_{2} t)\cos \gamma ] + z_{r} (\theta )\cos (\omega_{1} t)\sin \gamma + a\sin (\omega_{1} t) + b\cos (\omega_{1} t)\cos \gamma \hfill \\ z_{s} = - x_{r} (\theta )(\sin (\omega_{2} t)\sin \gamma ) - y_{r} (\theta )(\cos (\omega_{2} t)\sin \gamma ) + z_{r} (\theta )\cos \gamma + vt \hfill \\ \end{gathered} \right.$$

**Fig. 6:**
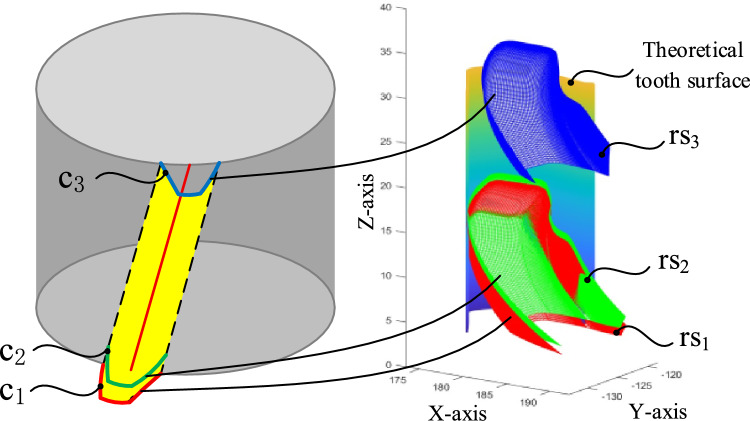
Sweeping trajectories of discrete curves on the flank face.

**Fig. 7:**
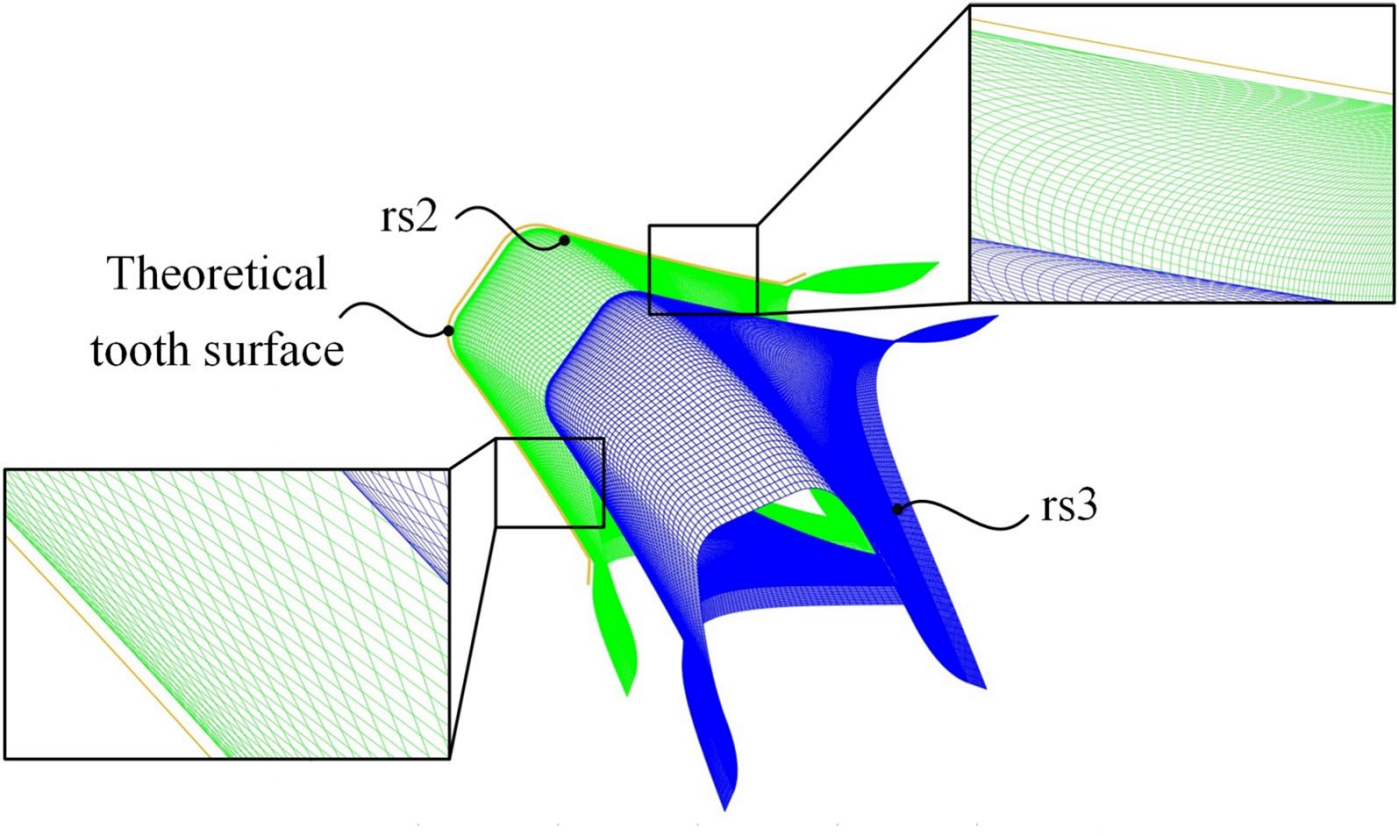
Interference in judgment.

By Eq. (29), the sweeping trajectories of three discrete curves on the flank face are examined as shown in Fig. 6. According to the tool design principle, the cutting edge, i.e. c_1_, is conjugate with the workpiece. Therefore, the sweeping trajectory of the cutting edge, i.e. rs_1_, is tangent to the theoretical tooth surface. The trajectories of the other parts on the flank face must be separate from the workpiece to avoid interference. For straight-tooth workpieces, this positional relationship can be examined through end-face projection. The end-face projection view in Fig. 7 provides a critical visual verification of the non-interference condition. It clearly shows that the sweeping trajectories of curves behind the cutting edge (rs_2_, rs_3_) lie entirely within the material region removed by the cutting edge’s trajectory (rs_1_), without intersecting the final theoretical tooth surface. This graphical proof is essential for validating that the designed helical flank face will not collide with the machined gear tooth during the skiving process, ensuring the feasibility of the tool geometry. It can be concluded that no interference occurred.

The sweeping trajectory method provides a computationally efficient approach for interference detection. While the mathematical model of Eqs. (25)-(29) appears complex, its actual implementation involves calculating the trajectories of only a few discrete representative curves on the flank face as shown in Fig. 6, rather than simulating the entire surface continuously. This discrete sampling strategy significantly reduces the computational burden. The analysis for a given tool geometry can be completed in seconds on a standard engineering computer, making it highly practical for iterative design and validation in an industrial setting. This efficiency is a key advantage over more computationally intensive full-scale multi-body dynamic simulations, allowing designers to quickly verify tool geometry before committing to physical prototyping.

## Analysis of offset skiving process parameters

### Offset gear skiving simulation

**Fig. 8:**
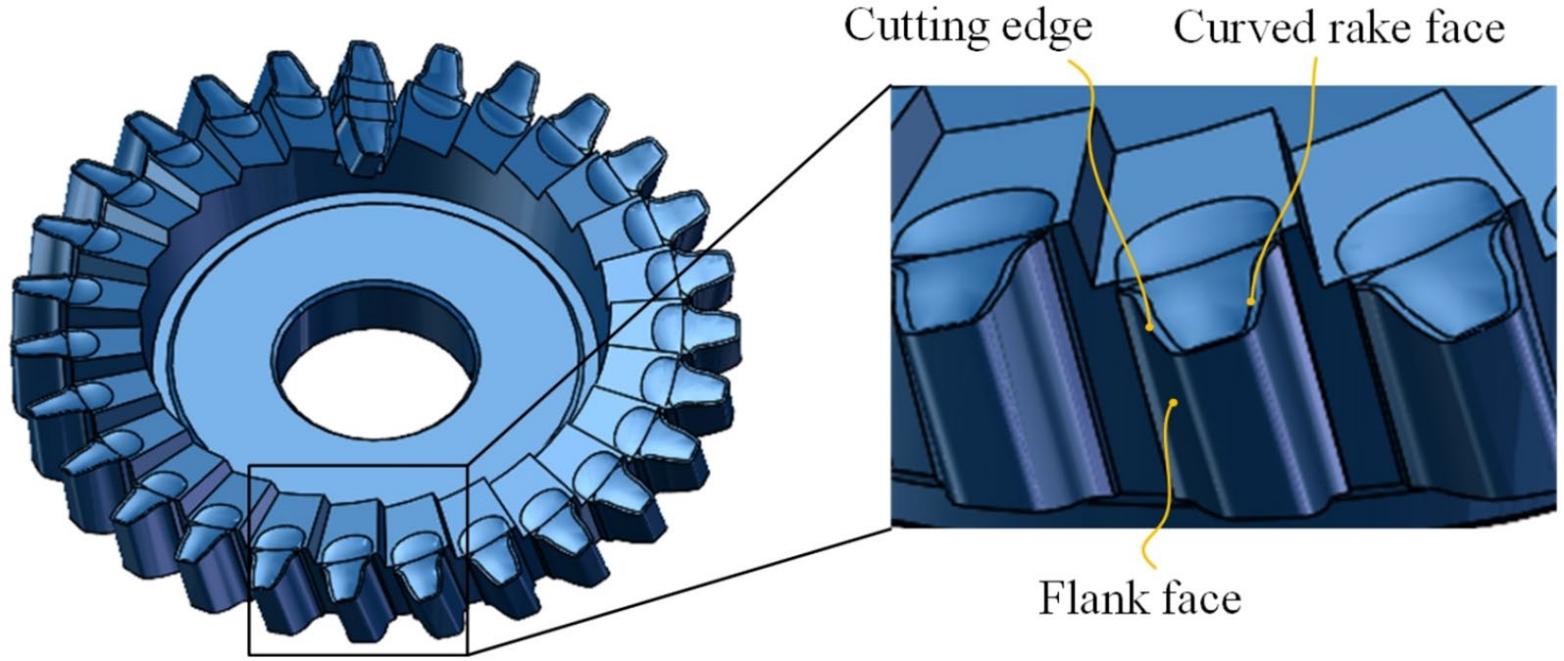
Geometric configuration and model of the cylindrical skiving tool.

In order to verify the effectiveness of the tool design method proposed in this paper, finite element modeling and analysis were carried out. Firstly, a three-dimensional model of the tool was established. For the workpiece shown in Table 1, the tool parameters as shown in Table 2 were selected, and the tool was designed according to the method described in Section "Cylindrical skiving tool design". The three-dimensional model of the tool is shown in Fig. 8.

**Table 1:** Workpiece parameters.

Number of teeth	Tip diameter (mm)	Root diameter (mm)	Pressure angle (°)	Module (mm)	Cross-bar distance (mm)
87	449.3	432.572	20	5.08	454.249

**Table 2:** Tool parameters.

Number of teeth	Working rake angle/(°)	Spiral angle/(°)	Tool diameter (mm)	Direction of rotation	Planes of section
27	8	18.5	146.996	Left	0

**Fig. 9:**
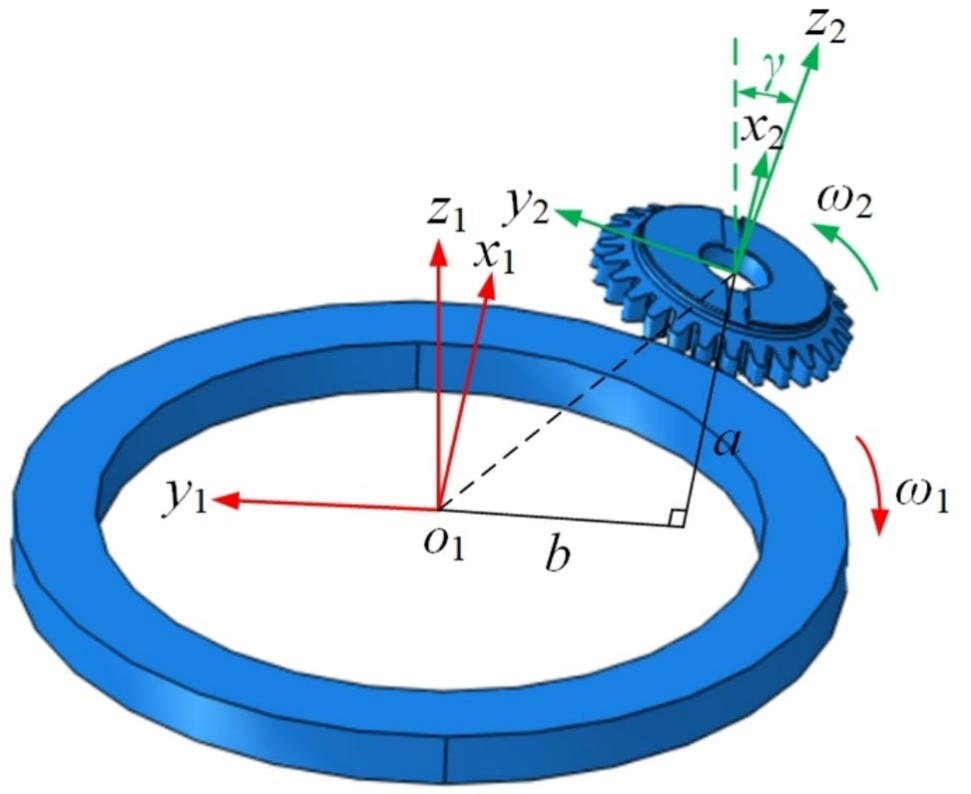
Skiving simulation model.

**Fig. 10:**
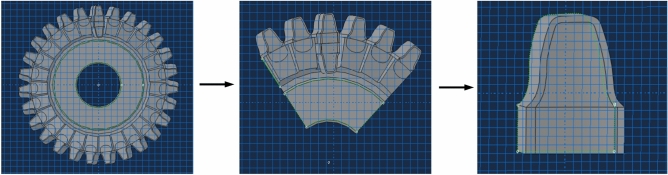
Simplified skiving tool simulation model.

The complete skiving simulation model is shown in Fig. 9. To reduce computational cost while preserving the essential cutting mechanics, the model was simplified to a single-tooth cutting configuration, as illustrated in Fig. 10. The cylindrical skiving tool and the workpiece are configured with a shaft intersection angle *γ*, a center distance *a* and a offset distance *b* between the tool and the workpiece, where *γ*=20°,* a* and *b* are adjusted to regulate the cutting depth. The cutting tool and the workpiece rotate in inverse proportion to the ratio of their tooth numbers.

The material of the workpiece is 25CrMo4, and its material parameters are listed in Table 3. The skiving tool was modeled as a rigid body, given that its deformation during the cutting of 25CrMo4 is negligible compared to the workpiece. The tool material was defined as tungsten carbide (hard metal), and its material parameters are listed in Table 4.

**Table 3:** 25CrMo4 material properties^17^.

Density (*kg*/*m*^3^)	Modulus of elasticity (*MP*_a_)	Poisson’s ratio (*v*)	Specific heat (*J*/(*kg*·*k*))	Thermal conductivity (*w*/(*m*·*k*))
7850	2.1×10^5^	0.269	450	47

**Table 4:** WC material properties.

Density (*kg*/*m*^3^)	Modulus of elasticity (*MP*_a_)	Poisson’s ratio (*v*)	Specific heat (*J*/(*kg*·*k*))	Thermal conductivity (*w*/(*m*·*k*))
15700	6.5×10^5^	0.25	470	59

Its constitutive equation^18^ is:

30 $$\overline{\sigma } = (A + B\varepsilon^{n} )(1 + c\ln \frac{{\mathop \varepsilon \limits^{ \cdot } }}{{\varepsilon_{0} }})\left[ {1 - (\frac{{T - T_{mo} }}{{T_{ml} - T_{mo} }})^{m} } \right]$$

Eq. (30) in which $\overline{\sigma }$ is the equivalent stress, *A* is the yield strength of the material, *B* and *n* are the strain intensification coefficients; *ε* is the equivalent plastic strain, *c* is the strain rate sensitivity coefficient; *m* is the temperature softening index;$\dot{\varepsilon }$ is the strain,$\varepsilon_{0}$ is the reference plastic strain rate, set to 1.0 s^-1^; *T*_m1_ is the melting temperature of the material and *T*_mo_ is the reference temperature. The values of each parameter in the Johnson-Cook constitutive model for 25CrMo4 material are listed in Table 5.

**Table 5:** Parameters of the Johnson-Cook constitutive model^17^.

$A(MP_{a} )$	$B(MP_{a} )$	$n$	$m$	$c$	$T_{ml}$	$T_{{{\mathrm{mo}}}}$
714	563	0.518	0.698	0.037	1600	25

The commercial finite element software ABAQUS/Explicit (version 2022) was used to conduct the coupled thermo-mechanical simulations. To accurately simulate the skiving process, the finite element model was established with specific settings and verifications. For chip formation and separation, a combination of a physical shear failure criterion and adaptive remeshing (ALE - Arbitrary Lagrangian-Eulerian formulation) available in ABAQUS was employed. The shear failure criterion was based on a critical equivalent plastic strain at the tool-chip interface. When the accumulated plastic strain in an element reached a critical value (set to 0.8 for 25CrMo4 based on preliminary tests and literature^18^), the element was considered to have failed and was allowed to separate from the workpiece matrix, facilitating chip formation. This approach, coupled with the ALE remeshing, avoids excessive mesh distortion and realistically simulates the continuous chip flow observed in skiving.

The boundary conditions were defined as follows: the workpiece was fixed in all degrees of freedom at its inner cylindrical surface, simulating a rigid clamping condition. The tool was assigned rotational and translational velocities to achieve the required cutting speed and feed motion. The tool-workpiece interaction was defined as a surface-to-surface contact, with a penalty friction formulation a coefficient of friction of 0.3.

**Fig.11:**
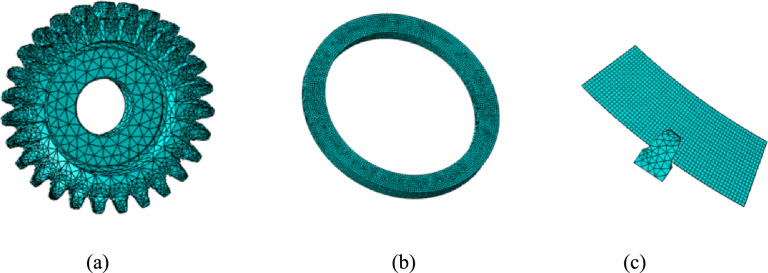
Mesh generation and model simplification.

The mesh configuration for both the tool and the workpiece is illustrated in Fig. 11. Regarding the element type, both the tool and the workpiece were meshed with linearly interpolated, reduced-integration, coupled temperature-displacement hexahedral elements. This element type is suitable for coupled thermo-mechanical analysis as it simultaneously calculates displacement and temperature fields, which is essential for accurately simulating the cutting process involving severe plastic deformation and heat generation.

A mesh sensitivity study was conducted to ensure the independence of the simulation results from the mesh size. Three models with progressively refined global element sizes (60 *μm*, 40 *μm*, and 25 *μm*) were compared based on the predicted steady-state cutting force. The results showed that the difference in cutting force between the 40 *μm* and 25 *μm* models was less than 2%. Therefore, the model with a global element size of 40 *μm* was selected for all subsequent simulations to achieve a balance between computational accuracy and efficiency. The region near the cutting edge was locally refined with an element size of approximately 15 *μm* to better capture high stress and temperature gradients.

### Orthogonal experiment

In order to analyze the influence of process parameters on cutting force and temperature in offset skiving, a orthogonal experiment was implemented, that has three factors:

Factor A: Tool rotational speed (rad/s)

Factor B: Workpiece feed rate (mm/s)

Factor C: Cutting depth (mm)

The parameter levels for each factor are listed in Table 6.

**Table 6:** Factor-level design for machining process.

Level	A: tool rotational speed (rad/s)	B: feed rate (mm/s)	C: cutting depth (mm)
1	83.73	0.5	0.5
2	104.72	1	1
3	125.6	1.5	1.5

An L_9_(3^3^) orthogonal array was adopted to balance analysis accuracy and efficiency, as shown in Table 7.

**Table 7:** Experimental scheme design.

Trial No.	Factor			Experimental scheme
	A	B	C	
1	1	1	1	A_1_B_1_C_1_
2	1	2	2	A_1_B_2_C_2_
3	1	3	3	A_1_B_3_C_3_
4	2	1	2	A_2_B_1_C_2_
5	2	2	3	A_2_B_2_C_3_
6	2	3	1	A_2_B_3_C_1_
7	3	1	3	A_3_B_1_C_3_
8	3	2	1	A_3_B_2_C_1_
9	3	3	2	A_3_B_3_C_2_

Following the above orthogonal experiment setting, finite element models were established by the method in Section "Offset gear skiving simulation". Time-dependent cutting forces along coordinate system S_1_, as well as the maximum cutting temperature on the skiving tool, were obtained as shown in Table 8.

**Table 8:** Mean/Maximum cutting forces and maximum cutting temperatures.

Trial No.	Mean cutting force/ (N)			Maximum cutting force /(N)			Maximum cutting temperatures (°C)
	*x*_1_	*y*_1_	*z*_1_	*x*_1_	*y*_1_	*z*_1_	
1	1083.88	634.37	1101.09	1636.95	4791.14	9458.43	294.968
2	1109.02	430.60	500.20	1996.03	3043.78	4688.72	151.996
3	1100.58	870.64	1802.48	2633.29	5127.42	11167.9	171.593
4	1748.24	532.90	357.56	3123.22	2551.77	4039.15	154.836
5	1713.695	909.84	1436.21	3123.22	5407.7	10605.3	171.349
6	1724.43	721.22	895.06	3129.28	4252.33	9481.96	268.158
7	2486.26	1057.35	1215.41	4493.32	5561.29	11020.1	170.588
8	2494.46	915.04	790.28	4505.24	4823.02	9496.2	180.674
9	2499.81	789.73	398.45	4493.32	3443.96	5186.29	167.452

### Cutting force analysis

**Fig. 12:**
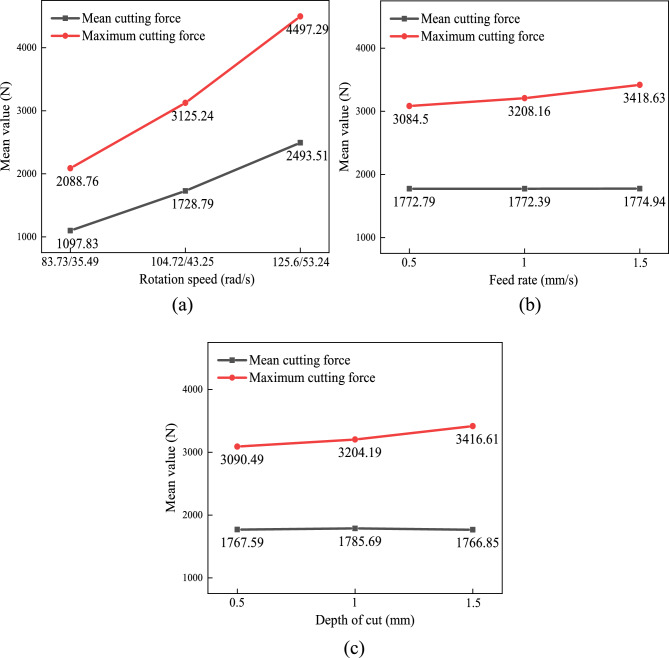
Trend of mean values of cutting force mean and maximum values of each factor.

To analyze the mean and maximum values of the *x*_1_-axis cutting force recorded in Table 8, range analysis^19^ was employed to evaluate the influence of different process parameters. The mean values of each parameter level and the range values of each factor are presented in Table 9, and the trends of the factor means are shown in Fig. 12.

**Table 9:** *x*_1_-axis cutting forces by factor.

Factor	Mean cutting force/(N)			Maximum cutting force/(N)		
	Rotation speed/(rad/s)	Feed rate (mm/s)	Depth of cut/(mm)	Rotation speed/(rad/s)	Feed rate (mm/s)	Depth of cut/(mm)
Mean value K_1_	1097.83	1772.79	1767.59	2088.76	3084.5	3090.49
Mean value K_2_	1728.79	1772.39	1785.69	3125.24	3208.16	3204.19
Mean value K_3_	2493.51	1774.94	1766.85	4497.29	3418.63	3416.61
Range R	-1395.68	-2.55	-18.84	-2408.54	-334.13	-326.12

Based on the range *R* values in Table 9, the factors influencing the mean and maximum cutting forces in cylindrical gear skiving tool are ranked as: feed rate > depth of cut > rotation speed. The feed rate has a significantly larger R value, indicating it most strongly affects cutting forces.

The observed influence hierarchy can be explained by the fundamental mechanics of cutting and the geometry of skiving. Feed rate exerts the dominant influence because it directly controls the undeformed chip thickness in the direction of tool motion. An increase in feed rate proportionally increases the cross-sectional area of the chip being removed per unit time, leading to a near-linear rise in the cutting force required to shear the material. Depth of cut primarily determines the width of the cutting edge in engagement. While increasing the depth of cut also enlarges the chip cross-section, its effect on the instantaneous cutting force is slightly less pronounced than that of the feed rate in this skiving configuration, as the cutting edge engages progressively. Rotation speed (cutting speed) has the least direct influence on the steady-state cutting force magnitude according to the range analysis. Although higher cutting speeds can induce strain-rate hardening in the workpiece material, this effect is often counterbalanced by thermal softening due to increased heat generation. Furthermore, the cutting speed does not alter the fundamental geometry of the uncut chip in the same direct way as feed and depth of cut. Its primary influence is on the cutting temperature, tool wear rate, and dynamic stability, rather than on the gross plastic deformation force, which aligns with the separate observation that rotation speed most significantly affects cutting temperature.

In the mean cutting force table, the optimal rotation speeds are 83.73 rad/s (tool) and 35.49 rad/s (workpiece). The optimal feed rate is 1 mm/s, and the optimal depth of cut is 1.5 mm. For maximum cutting force, the optimal rotation speeds remain 83.73 rad/s (tool) and 35.49 rad/s (workpiece), with a feed rate of 0.5 mm/s and depth of cut of 0.5 mm.

### Cutting heat analysis

**Fig. 13:**
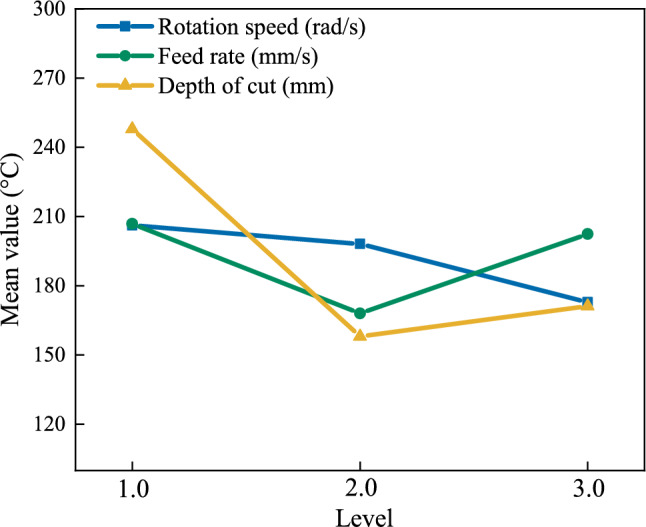
Mean value plot of cutting heat across process parameter levels.

In order to analyze the combined effect of process parameters on cutting heat, an orthogonal experiment was designed. data from Table 8 yielded level mean values. The range analysis method was used to analyze the influence of each process parameter on the maximum cutting temperature. The analysis results were recorded in Table 10, and the average value diagrams of the cutting heat factors levels are shown in Fig. 13.

**Table 10:** Thermal analysis of cutting parameters.

Factor	Rotation speed/(rad/s)	Feed rate/(mm/s)	Depth of cut/(mm)
Mean value K_1_	206.19	206.8	247.93
Mean value K_2_	198.11	168.01	158.09
Mean value K_3_	172.9	202.4	171.18
Range R	-33.28	-38.79	-89.84

The range *R* values indicate that rotation speed most significantly affects maximum cutting temperature, followed by feed rate and depth of cut. To minimize cutting temperature, optimal levels are: tool speed 125.6 rad/s, workpiece speed 53.24 rad/s, feed rate1 mm/s, and depth of cut 1 mm.

In summary, the synergistic effects of multiple process parameters reveal that feed rate exerts a more pronounced influence on cutting force compared to rotation speed and depth of cut. To mitigate cutting force during machining, priority should be given to controlling the offset tooth feed rate. Concurrently, the analysis of parameter impacts on cutting temperature demonstrates the following hierarchy: rotation speed > feed rate > depth of cut. An optimal parameter combination was identified to minimize maximum cutting temperature under specified conditions.

## Comparative analysis of cutting performance between curved-rake-face cutter and plane-rake-face cutter

### Analysis of the skiving process

**Fig. 14:**
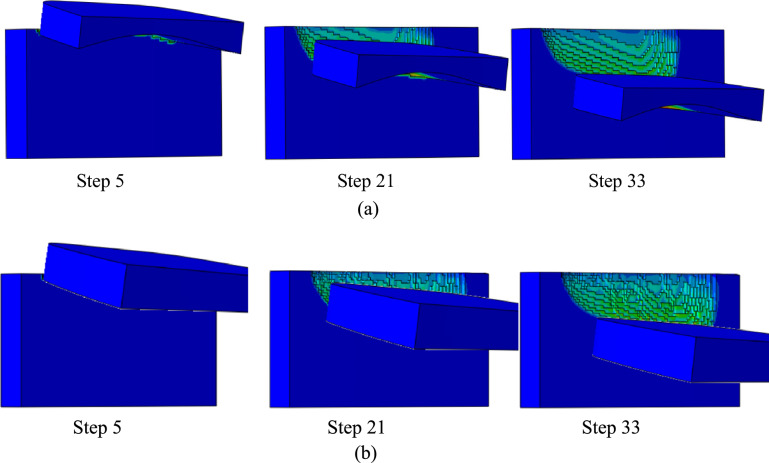
Simplified stress cloud diagrams for the skiving process.

Finite element post-processing in ABAQUS selected simulation states at steps 5, 21, and 33, as shown in Fig. 14. The stress nephograms reveal the evolution of the tool-workpiece contact and stress distribution as the cutting progresses from initial engagement (step 5) through intermediate (step 21) to a later stage (step 33). Qualitative observation of Fig. 14 indicates a difference in the stress distribution pattern between the two tool designs. For the curved-rake-face cutter, the high-stress region (shown in red) appears to be more confined to the immediate vicinity of the cutting edge and maintains a relatively consistent spatial extent across the three steps. This suggests a more contained and steady load application. For the plane-rake-face cutter, the high-stress region appears somewhat more diffuse and exhibits a slightly more variable shape and distribution along the cutting edge at different steps. This visual difference aligns with the quantitative force and temperature results presented in subsequent sections, which show that the curved-rake-face cutter leads to lower force fluctuations and a more uniform thermal distribution, contributing to enhanced process stability.

### Analysis of cutting force

To validate the cylindrical gear skiving tool design based on the surface conjugation principle, a comparative analysis of cutting forces, thermal distribution, and machining stability was conducted between curved-rake-face gear skiving tool and plane-rake-face gear skiving tool.

**Fig. 15:**
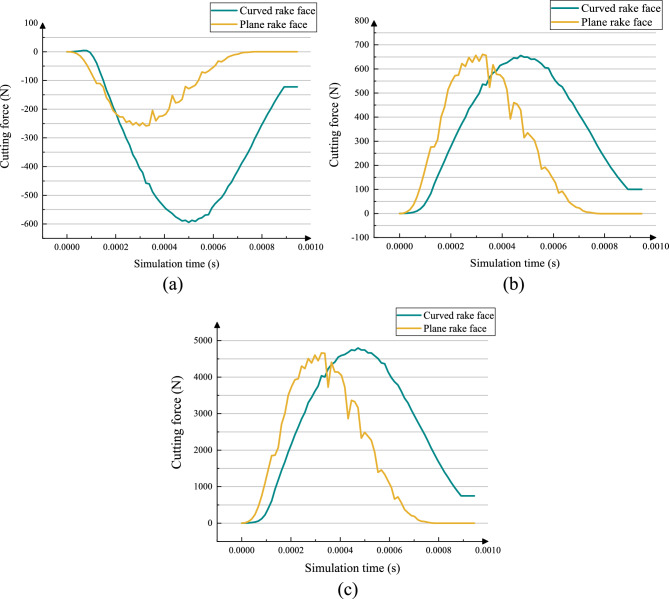
Comparison results of the two tool types.

As shown in Fig. 15, the cutting forces for both tools along the *x*_1_-, *y*_1_-, and z_1_-axes are compared. Under identical machining parameters, both tools exhibit stable cutting force trends. As shown in Fig. 15(a), the direction of axis *x*_1_ cutting forces for both tools increase rapidly from zero, stabilizing into a fluctuating stage after approximately 0.0001 s. Notably, the curved-rake-face cutter demonstrates significantly lower fluctuation amplitude than the plane-rake-face cutter, with a smaller peak-to-valley difference. It is observed that the peak cutting force in the *x*_1_ direction for the curved-rake-face cutter (≈600 N) is higher than that of the plane-rake-face cutter (≈250 N). This specific increase can be attributed to the fundamental change in tool geometry. The designed curved rake face, optimized to maintain a uniform working rake angle along the entire cutting edge under offset conditions, alters the direction of the resultant cutting force vector compared to the plane rake face. In the *x*_1_ direction, the curved cutter geometry leads to a more direct radial force component during chip formation. This is a characteristic trade-off of the design: achieving uniform rake angles and superior stability in other performance metrics may result in a redistribution of force components among different axes.

As shown in Fig. 15(b), the direction of axis *y*_1_ cutting forces exhibit a similar pattern to the *x*_1_-axis: a rapid initial rise followed by stabilized fluctuations. However, the curved-rake-face cutter displays more gradual force fluctuations and a slightly lower overall force magnitude, approximately 650 N lower, than the plane-rake-face cutter. The plane cutter exhibits significantly larger force oscillations due to rigid impact.

For the direction of axis *z*_1_ cutting force, representing the principal cutting force which dominates the total machining power, the forces in the other axes are relatively small. Consequently, its trend reflects the overall cutting force. Fig. 15(c) reveals a critical trade-off. The curved-rake-face cutter exhibits a marginally higher peak principal force, approximately 4800 N, than the plane-rake-face cutter, approximately 4600 N. This slight increase in peak force would lead to a negligible rise in instantaneous power consumption, as power is linearly related to force. However, the most significant advantage of the curved cutter lies in its dramatically lower force fluctuation amplitude.

This combination of characteristics—a slightly higher peak force but greatly reduced fluctuations—has distinct implications for tool wear and power demand. The large, cyclic fluctuations generated by the plane-rake-face cutter impose severe impact loading and fatigue stress on the cutting edge, which is a primary mechanism for micro-chipping, premature coating delamination, and accelerated tool wear. In contrast, the stable cutting force provided by the curved-rake-face cutter ensures a smooth, predictable load on the cutting edge. This stability significantly mitigates fatigue-based wear mechanisms, thereby extending tool life. The benefit of dramatically improved tool longevity far outweighs the minimal penalty in peak power demand. Furthermore, a stable cutting process often translates to consistent power draw, which is beneficial for overall machine tool energy management and process reliability.

Therefore, selecting the curved-rake-face cutter enhances tool longevity and process stability, which is paramount for industrial efficiency.

### Analysis of cutting temperature

**Fig. 16:**
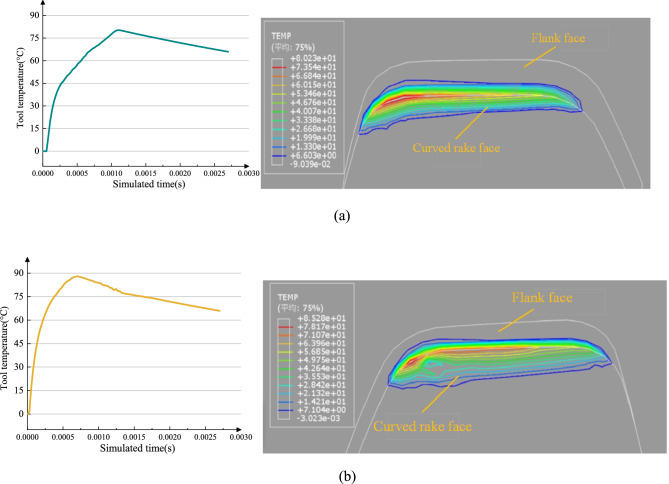
Temperature curves and isotherm contour maps of rake faces for different tools.

The cutting temperature curves and isotherm contour maps for the curved-rake-face cutter and plane-rake-face cutter are shown in Fig. 16. The temperature was measured at the tool-chip interface near the tool tip, as this region experiences the most severe thermomechanical loading and is critical for assessing tool performance.

As shown in Fig. 16(a) for the curved-rake-face cutter, the high-temperature region (indicated by red/orange colors) is concentrated near the tool tip, with a peak of approximately 80°C. Critically, the isothermal lines in this region and across the rake face are relatively uniformly and widely spaced. This pattern indicates a gradual thermal gradient, suggesting efficient heat conduction and a reduced risk of localized thermal stress. In contrast, for the plane-rake-face cutter (Fig. 16(b)), the high-temperature zone is more extensive and spreads rearward, with a slightly higher peak of about 85°C. More importantly, the isothermal lines, particularly in the transition zone between the hot spot and the cooler tool body, are visibly more crowded and densely packed. This densely packed isotherm pattern unequivocally reveals a steeper thermal gradient compared to the curved-rake-face cutter, implying a more abrupt temperature change and a higher propensity for thermal stress concentration.

In terms of thermal dynamics, the curved-rake-face cutter exhibits a narrower temperature fluctuation range, indicating steady heat input and high thermal stability during machining. Conversely, the plane-rake-face cutter shows pronounced temperature oscillations, revealing significant thermal shock. This difference arises because the curved cutter’s optimized structure improves heat distribution, reducing the peak temperature by approximately 20°C while decreasing temperature fluctuation amplitude by 50%. This effectively mitigates thermal fatigue risk, extends tool life, and preserves material cutting performance. These findings have direct implications for tool cooling and coating strategies. The concentrated heat zone in the curved-rake-face cutter suggests that cooling strategies can be targeted more effectively at the tool tip for maximum efficiency. Furthermore, the overall lower and more stable temperatures reduce the demand on the tool coating’s thermal resistance, potentially allowing for the use of a wider range of coating materials optimized for wear resistance rather than extreme heat protection. In contrast, the extensive heat accumulation and sharp gradients observed in the plane-rake-face cutter necessitate coatings with superior thermal barrier properties and a robust cooling system to mitigate thermal cracking and coating delamination. Thus, the thermal data further validates the comprehensive thermal management advantages of the curved-rake-face cutter, providing strong evidence for enhancing gear skiving efficiency and tool durability.

### Analysis of stress states on the workpiece

A comparative analysis is performed on the stress states of the tooth surfaces machined by the cutting tools with curved rake face and plane rake face along the directions of axes *x*_1_, *y*_1_, and *z*_1_.

**Fig. 17:**
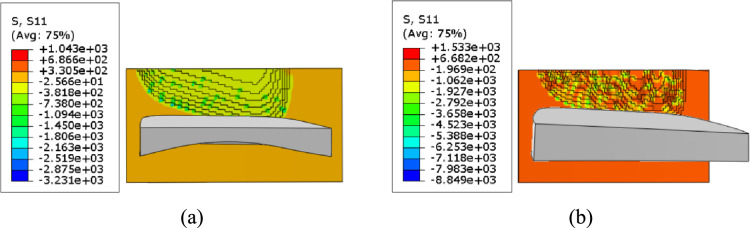
Stress of the machined tooth surface along axis *x*_1_.

As shown in Fig. 17, along axis *x*_1_, the tooth surface machined by curved-rake-face cutter exhibits a gradient arc-shaped stress distribution, the maximum tensile stress was 1043 MPa. Compressive stresses accounted for 85% of the total. In contrast, the tooth surface machined by plane-rake-face cutter demonstrates a steep stress concentration peak, the maximum tensile stress was 1533 MPa, which was 47% higher than that of the tooth surface machined by curved-rake-face cutter. Compressive stresses accounted for 77% of the total.

**Fig. 18:**
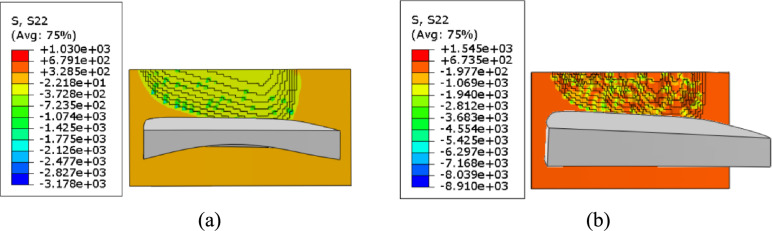
Stress of the machined tooth surface along axis *y*_1_.

As shown in Fig. 18, along axis *y*_1_, the tooth surface machined by curved-rake-face cutter with stresses evenly diffusing toward the tooth surface, the maximum tensile stress was 1030 MPa. Compressive stresses accounted for 85% of the total. Conversely, the tooth surface machined by plane-rake-face cutter is characterized by a single-peak narrow-band concentration defect, exhibiting the maximum tensile stress was 1545 MPa, which was 50% higher than that of the tooth surface machined by curved-rake-face cutter. Compressive stresses accounted for 77% of the total.

**Fig. 19:**
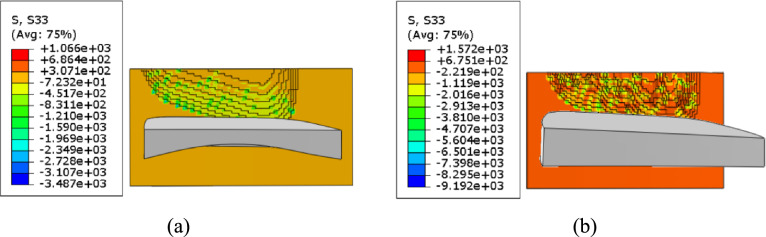
Stress of the machined tooth surface along axis *z*_1_.

As shown in Fig. 19, along axis *z*_1_, the tooth surface machined by curved-rake-face cutter achieves smooth stress flow, the maximum tensile stress was 1066 MPa. Compressive stresses accounted for 85% of the total. In stark contrast, the tooth surface machined by plane-rake-face cutter accompanied by attenuated central compressive strength and annular tensile stress proliferation bands along the periphery that promote potential fatigue cracks. The maximum tensile stress was 1572 MPa, which was 47% higher than that of the tooth surface machined by curved-rake-face cutter. Compressive stresses accounted for 77% of the total.

As shown in Table 11, a clear summary is provided for the key stress values presented in Figs. 17-19, facilitating a direct comparison between the two tool designs.

**Table 11:** Summary of stress characteristics.

Stress direction	Tool type	Max tensile stress (MPa)	Increase	Percentage of compressive stress (%)
Along axis *x*_1_	Curved-rake-face	1043	-	85
	Plane-rake-face	1533	+47%	77
Along axis *y*_1_	Curved-rake-face	1030	-	85
	Plane-rake-face	1545	+50%	77
Along axis *z*_1_	Curved-rake-face	1066	-	85
	Plane-rake-face	1572	+47%	77

(Note: The "-" in the table indicates the baseline for comparison).

In conclusion, the cutting tool with curved rake face can achieve smaller tensile stress, more uniform stress distribution, and a higher proportion of compressive stress.

## Gear skiving experiment

### Experiment process

**Fig. 20:**
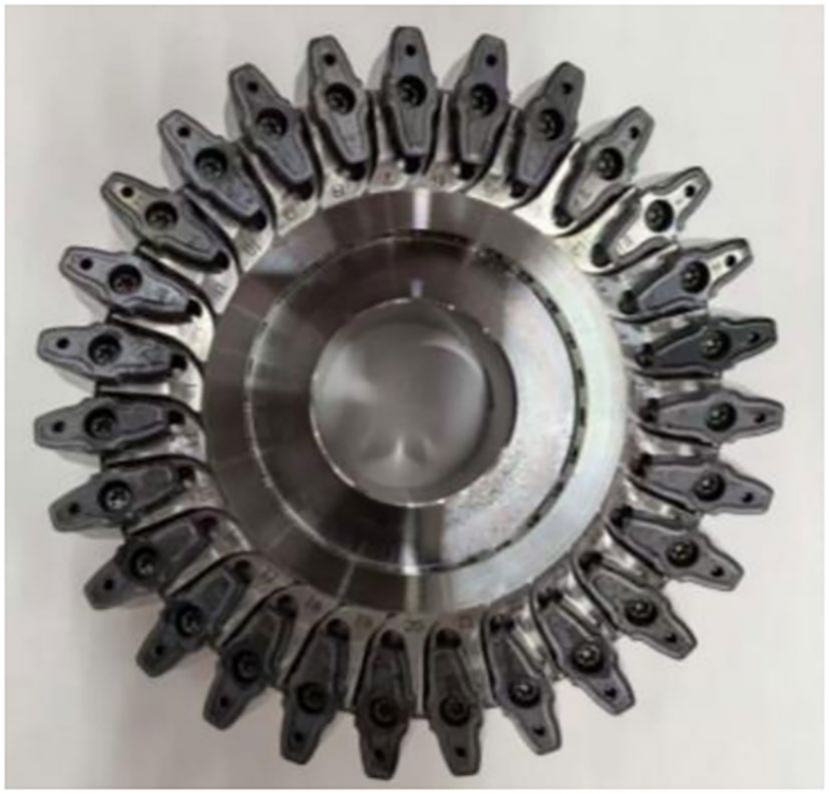
Cylindrical gear skiving tool.

To verify the correctness and manufacturability of the tool design, an experiment was conducted. A skiving cutter with indexable inserts was fabricated, as shown in Fig. 20. The fabrication of the proposed curved-rake-face cutter involved the following key steps to realize its complex geometry:

1. Insert Blank Preparation: Tungsten carbide blanks were ground to the basic wedge shape and precise overall dimensions.
2. Free-form Rake Face Grinding: The core geometry—the designed free-form curved rake face—was generated on a 5-axis CNC tool grinder using a precisely profiled diamond grinding wheel. The grinding wheel profile was dressed according to the inverse of the tool’s rake face geometry data, which was directly output from the design algorithm described in Section "Curved rake face". The CNC program was generated from the same design coordinates, ensuring accurate realization of the uniform working rake angle.
3. Helical Flank Face Grinding: The cylindrical helical flank face was ground in a subsequent setup on the same 5-axis grinder. The insert was mounted at the specified helix angle, and a standard diamond grinding wheel was used to generate the helical surface by coordinating rotational and linear axes movements.
4. Finishing and Coating: After grinding, the cutting edges were honed with a micro-blasting process to remove sharp burrs and improve chipping resistance. Finally, a multi-layer TiAIN coating was applied via Physical Vapor Deposition to enhance wear resistance and reduce friction.

**Fig. 21:**
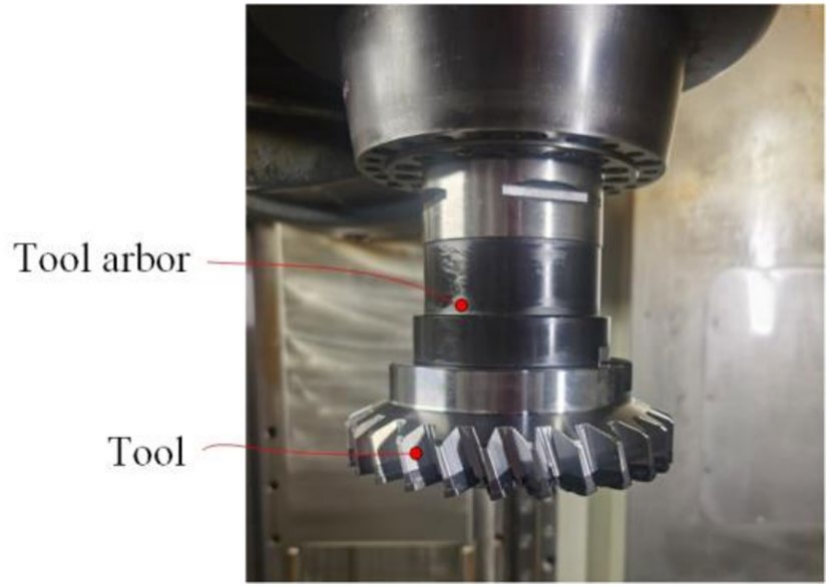
The clamped cutting tool.

The finished inserts were then assembled onto a dedicated tool body to form the complete skiving cutter, as shown in Fig. 20. In a gear skiving machine tool, as shown in Fig. 21, the workpiece was machined by this cutter under the processing parameters shown in Table 12.

**Table 12:** Practical machining parameters.

	Number of radial feeds	Depth of cut /(mm)	Axial feed /(mm/r)	Workpiece speed /(r/min)
Roughing	1-10	0.75	0.3	500
Semi-finishing	11	0.5	0.1	500
Finishing	12	0.3	0.05	500

**Fig. 22:**
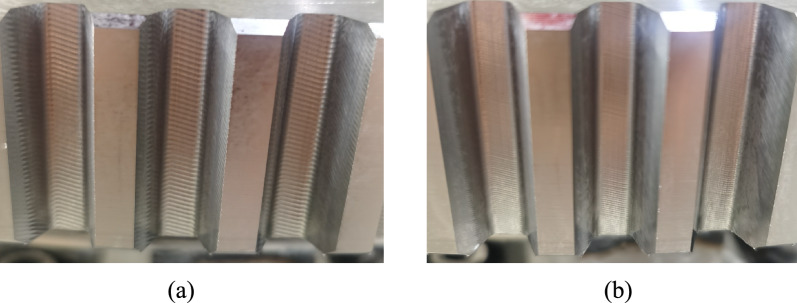
Tooth surface after machining.

During the cutting process of cylindrical gear skiving tool, the vibration of the machine tool was normal, and there was no violent vibration caused by interference. The cutting marks were uniform and clear, as shown in Fig. 22, proving the stability of the processing procedure. At relatively high rotational speeds of the workpiece and the tool, a high processing efficiency was achieved, where the processing time was controlled within 25 minutes.

### Analysis of experiment results

The machined workpiece was detected at a gear measurement center, including tooth profile accuracy, pitch error and lead error, as shown in Appendix A.

#### Tooth profile accuracy

The tooth profile accuracy met the GB/T 7th-grade standard, exceeding the workpiece’s required accuracy. The primary deviations were as follows:

Maximum positive deviation: +8.2 *μm* (occurring at the tooth tip)

Maximum negative deviation: -6.5 *μm* (occurring near the tooth root)

Considering the positioning error of the mechanically clamped insert, this level of experimental accuracy confirms the correctness of the tool design method proposed in this paper.

#### Pitch and lead accuracy

The inspection data demonstrated compliance with the GB/T 8th-grade standard. The key measurements are summarized in Table 13.

**Table 13:** Summary of key gear inspection results.

Inspection item	Flank	Value (*μm*)	GB/T Grade
Cumulative pitch error	Left	21.3	8th
	Right	21.3	8th
Lead error	Left	12.8	8th
	Right	14.2	8th

The cumulative pitch error was measured at 21.3 *μm*, with a uniform single-pitch error distribution, indicating good feeding stability. The lead errors for the left and right flanks were 12.8 *μm* and 14.2 *μm*, respectively. These results indicate that the clamping accuracy of the workpiece and the tool could be further improved.

The tool trial-cut process and the workpiece inspection report mentioned above indicated that the tool design method proposed in this paper can produce non-interfering and low-cost cylindrical skiving tools. The design of curved rake face improves the tool’s cutting performance, enabling smoother cutting and higher processing efficiency.

## Conclusions

To address the limitations of traditional gear skiving tool design, including precision degradation after regrinding and inadequate machining stability, this paper proposes a novel design method for cylindrical gear skiving tool with uniform working rake angle based on origin offset. Conclusions were drawn through comprehensive research employing theoretical modeling, numerical simulation, and cutting experiments.

1. A motion model for offset gear skiving is established, and the conjugate relationship between the cutting tool and the workpiece is derived.
2. The cutting tool is constructed by a free-form rake face and a helical flank face. The working rake angle is controlled to be uniform, and the flank face is confirmed to have no interference through sweeping trajectory calculation.
3. A multi-physics coupling simulation model for cutting forces and temperature field is developed by finite element method. The influence of process parameters on cutting force and temperature is analyzed. Feed rate exhibits the most significant influence on cutting force, while rotational speed dominates the impact on cutting temperature.
4. The cutting performance of the designed skiving tool with curved rake face is compared with that of the cutting tool with plane rake face. The curved-rake-face cutter reduces cutting force fluctuation amplitude and mitigates cyclic impact loading. Additionally, it achieves a more uniform temperature distribution, and lowers the maximum cutting temperature by approximately 15% compared to the plane-rake-face cutter. Moreover, the cutting tool with curved rake face can achieve smaller tensile stress, more uniform stress distribution, and a higher proportion of compressive stress.
5. A cutting experiment on the actual product was conducted to verify the correctness and effectiveness of the tool design method proposed in this paper. The design of curved rake face improves the cutting tool’s cutting performance and enables smoother processing.

## Data Availability

All data generated or analysed during this study are included in this published article.

## Appendix A

Test report on the accuracy of test cut workpieces.
